# Effect of Loading and Functionalization of Carbon Nanotube on the Performance of Blended Polysulfone/Polyethersulfone Membrane during Treatment of Wastewater Containing Phenol and Benzene

**DOI:** 10.3390/membranes10030054

**Published:** 2020-03-24

**Authors:** Mabusha S. Rameetse, Oluseyi Aberefa, Michael O. Daramola

**Affiliations:** 1 School of Chemical and Metallurgical Engineering, Faculty of Engineering and Built Environment, University of the Witwatersrand, Johannesburg, Private Bag 3, Wits 2050, South Africa; 1448424@students.wits.ac.za (M.S.R.); seyiaberefa@yahoo.com (O.A.); 2 Department of Chemical Engineering, Faculty of Engineering, Built Environment and Information Technology, University of Pretoria, Private bag X20 Hatfield, Pretoria 0028, South Africa

**Keywords:** carbon nanotubes, membranes, phenol, benzene, functionalization

## Abstract

In this study, a carbon nanotube (CNT)-infused blended polymer membrane was prepared and evaluated for phenol and benzene removal from petroleum industry wastewater. A 25:75 (by weight %) blended polysulfone/polyethersulfone (PSF/PES) membrane infused with CNTs was prepared and tested. The effect of functionalization of the CNTs on the quality and performance of the membrane was also investigated. The membranes were loaded with CNTs at different loadings: 0.5 wt. %, 1 wt. %, 1.5 wt. % pure CNTs (pCNTs) and 1 wt. % functionalized CNTs (fCNTs), to gain an insight into the effect of the amount of CNT on the quality and performance of the membranes. Physicochemical properties of the as-prepared membranes were obtained using scanning electron microscopy (SEM) for morphology, Raman spectroscopy for purity of the CNTs, Fourier transform infrared (FTIR) for surface chemistry, thermogravimetric analysis (TGA) for thermal stability, atomic force microscopy (AFM) for surface nature and nano-tensile analysis for the mechanical strength of the membranes. The performance of the membrane was tested with synthetic wastewater containing 20 ppm of phenol and 20 ppm of benzene using a dead-end filtration cell at a pressure ranging from 100 to 300 kPa. The results show that embedding CNTs in the blended polymer (PSF/PES) increased both the porosity and water absorption capacity of the membranes, thereby resulting in enhanced water flux up to 309 L/m^2^h for 1.5 wt. % pCNTs and 326 L/m^2^h for 1 wt. % functionalized CNT-loaded membrane. Infusing the polysulfone/polyethersulfone (PSF/PES) membrane with CNTs enhanced the thermal stability and mechanical strength. Results from AFM indicate enhanced hydrophilicity of the membranes, translating in the enhancement of anti-fouling properties of the membranes. However, the % rejection of membranes with CNTs decreased with an increase in pCNTs concentration and pressure, while it increased the membrane with fCNTs. The % rejection of benzene in the pCNTs membrane decreased with 13.5% and 7.55% in fCNT membrane while phenol decreased with 55.6% in pCNT membrane and 42.9% in the FCNT membrane. This can be attributed to poor CNT dispersion resulting in increased pore sizes observed when CNT concentration increases. Optimization of membrane synthesis might be required to enhance the separation performance of the membranes.

## 1. Introduction

The petroleum industry is vital to the sustainability of energy and economy of the globe and accounts for a large percentage of the world’s energy consumption. However, it also poses a serious threat to the environment with the vast amount of wastewater produced daily. The wastewater needs to be treated to meet the Environmental Protection Agency regulatory standards before disposing to the environment or being reused, and membrane-based treatment could be an option. Therefore, the availability of dependable membrane materials for the treatment of produced water from petroleum production could be instrumental to developing a point-of-use membrane system for use in this industry. This introduced the use of a polymeric membrane. However, due to their limitations, various membrane modification methods, such as polymer blending, have been explored. Polymer blending is a time- and cost-effective technique used to develop materials with unique anticipated properties depending on the type of membrane needed [1]. Advantages of polymer blending include, increased toughening, extended service temperature range, improved barrier properties and flame-retardant properties. Polyethersulfone (PES) and polysulfone (PSF) are reported to be widely used for water treatment purposes due to their good membrane-forming performances, outstanding heat stability, visual transparency, excellent solubility and selectivity as compared to other polymer counterparts. They are easy to fabricate, cost-effective and widely available in commercial markets [2]. However, they are both known to be hydrophobic, resulting in membrane fouling and insufficient mechanical strength, hence their application of membranes in various industries to treat wastewater remains limited [2]. This problem can be solved by embedding nanoparticles with hydrophilic properties, such as carbon nanotubes (CNTs).

Ever since the discovery of CNTs by Sumio Iijima in 1991, special attention has been centered on studying and understanding their structure, properties and use. They have strengthened the application of membrane technology, especially for water treatment purposes, due to their unique and outstanding characteristics. Some of their unique characteristics include the smoothness of their internal walls, as they result in low levels of friction with water molecules during filtration applications [3]. Recently, the application of CNT-filled membranes as oil-containing water separation materials has attracted wide-ranging attention due to their properties, which include low density, high porosity, electrical and thermal conductivity [4,5]. Other properties include tensile strength, higher elastic modulus and strain to fracture, the capability to endure twisting together with cross-sectional distortions and high compression without breakage [6]. These properties stabilize and strengthen membranes; they also enhance their separation performance. However, to increase the hydrophilicity of the membranes, CNTs need to be functionalized to improve the surface property of the membrane. Functionalization also improves the dispersion of CNTs in the polymer matrix during fabrication to obtain composites with intrinsic properties. This step is important because the more hydrophilic a membrane is the better its fouling resistance. In fact, most fouling agents are naturally hydrophobic [7].

A previous study was conducted where PSF/PES blended membranes were prepared at different compositions (0% PSF:100% PES, 100% PSF:0% PES, 20% PSF:80% PES, 25% PSF:75% PES, 50% PSF:50% PES and 80% PSF:20% PES), characterized and evaluated for the treatment of wastewater containing benzene and phenol. From these characterization and separation performance results, the best performing membrane (25% PSF:75% PES) was selected and further modified in this study [8]. In this study, five blended 25% PSF:75% PES membranes were prepared. From the five membranes, three were embedded with pure CNTs at different wt. % loading and one with functionalized CNTs. The objective of this study was to investigate the effect of CNT loading and functionalization of CNTs on the physicochemical properties, thermal stability, mechanical strength and separation performance of PES/PSF blended membrane during the treatment of phenol-containing wastewater. 

## 2. Materials and Methods 

### 2.1. Materials

The materials that would be required for this investigation are polysulfone (PSF) pellets (average molecular weight: 35,000 Da) from Sigma Aldrich, Johannesburg, South Africa; polyethersulfone (PES) crystal (0.025 mm × 150 mm × 150 mm) from GIC Scientific; n-methyl-2-pyrrolidone (NMP) 99%; from ACE chemicals, Johannesburg, South Africa); phenol (molecular biology) and benzene (≥99.7%), both from Sigma Aldrich, South Africa. Multi-walled CNTs with an outer diameter of about 10 nm and length of about 3–6 μm with a purity of 98% were obtained from Sigma Aldrich, South Africa; 98% H_2_SO_4_ and 65% HNO_3_ both from Sigma Aldrich, South Africa.

### 2.2. Preparation of Blended PSF/PES Membrane

Ten percent (polymer to solvent ratio) of 25% PSF:75% PES (by weight) were dissolved in n-methyl-2-pyrrolidone (NMP) under continuous agitation at room temperature for 12 h. The casting solution was cast using a casting blade set at 180 µm on smooth glass. The glass, together with the dope, was then immersed in distilled water for the phase separation step. The formed membranes were left to dry at room temperature for 24 hours. The membranes were then characterized.

### 2.3. Purification and Functionalization of CNTs

For purification, 1 g of raw CNTs were added to 100 mL of hydrochloric acid and then ultrasonicated for 4 h at 40 °C. The centrifuged residue was then washed with distilled water again and filtered using vacuum filtration until the pH of the filtrate was neutral. The filtered CNTs were then dried at 100 °C for 24 h.

For functionalization, 1 g of pure CNTs was reacted with a mixture of 75 mL of sulfuric acid (H_2_SO_4_) and 25 mL of nitric (HNO_3_); the mixture was ultrasonicated for 4 hours and heated at 40 °C. The same protocol for washing and drying used during purification was applied. The dried CNTs were then characterized to confirm successful functionalization using Fourier transform infrared spectroscopy (FTIR) to check the presence of functional groups.

### 2.4. Preparation of CNT/Blended Membranes

The membranes were synthesized using the phase inversion method, with pure CNTs at different concentrations of 0.5, 1.0 and 1.5 wt. % and one concentration of 1 wt. % of functionalized CNTs. For each membrane, the CNTs were dissolved in 15 mL of NMP and 10 wt. % of 25:75 PSF/PES dissolved in another 15 mL of NMP and stirred for 20 hours using a magnetic stirrer. After 20 h, the polymer solution was added to the CNT solution. The mixture was then stirred for 4 h until the CNTs had completely dissolved in the polymer mixture to form a casting solution. The casting solution was then cast using a casting blade set at 180 µm to form the polymeric nanocomposite membrane. The casted solution was then immersed in distilled water for the phase separation step, which takes a few seconds. The cast solution was left in the distilled water for 30 seconds until the membrane was formed. The formed membranes were left to dry at room temperature for 24 h to ensure the adequate loss of moisture before use.

### 2.5. Equilibrium Water Content (EWC) and Porosity

EWC is directly related to porosity. It measures the water absorption ability of a membrane by the pores. It was calculated using Equation (1), where *W*_w_ is the weight of the membrane when wet while *W*_d_ is the dry weight of the membrane.

(1) $$\mathrm{EWC}\;=\;\frac{Ww-Wd}{Ww}\times 100\%$$

Porosity of the membrane was determined by the mass loss of wet membrane after drying up. It was calculated using Equation (2), where ρ is the density of water and *V* is the total volume of the membrane.

(2) $$\mathrm{Porosity}\;=\;\frac{Ww-Wd}{\rho \times v}\;100\%$$

### 2.6. Membrane Characterization

Thermogravimetry analysis (TGA) was used to investigate the thermal degradation of the prepared membranes measurements which were carried out under nitrogen atmosphere in the temperature range from 25 to 800 °C and at a flow rate of 50 °C.

A nano-tensile tester was used to analyze the mechanical strength of the membranes. Each membrane was cut into 10 × 30 mm^2^ dimensions and tested. The force applied at membrane breakage and the difference between the initial length and length at the break for each sample were obtained. From these parameters, the tensile strength and Young’s modulus values were calculated using Equations (3) and (4), respectively.

(3) $$\mathrm{Tensile}\;\mathrm{Strength}\;=\;\frac{\mathrm{Force}}{\mathrm{Area}}$$

(4) $$\mathrm{Young}’s\;\mathrm{Modulus}\;=\;\frac{\mathrm{Tensile}\;\mathrm{Strength}}{\mathrm{Strain}}$$

Atomic force microscopy (AFM) was used to investigate the surface topography and roughness of the membranes. The microscope was operated in non-contact mode at a resonance frequency between 75 and 100 kHz and 1µm×1µm images were obtained. A Scanning Electron Microscope (SEM) was used to analyze the morphology of the membrane from the surface to the cross-section. The prepared samples were coated with carbon. The surface view and the cross-sectional view of the membranes were examined at magnification of 5000× at an acceleration voltage of 10 kV.

### 2.7. Membrane Performance

The separation performance of the membrane was assessed through the dead-end filtration method but firstly a standard experiment was conducted for pure water permeation to obtain the initial influx of the membrane and compare with that of wastewater separation. The flux was calculated using Equation (5), where *J*_w_ represents pure water flux (L/m^2^h), *V* is the volume of water (L) that permeated through the membrane, A is the area of the membrane (50 m^2^) while t stands for water permeation time (0.3 h). This separation system uses nitrogen gas to sustain the essential pressure gradient to force the feed solution into the membrane. The performance of the membrane was tested with synthetic wastewater containing 20 ppm of phenol and 20 ppm of benzene. The reason for using synthetic phenol and benzene containing wastewater is because produced water contains many contaminants. The choice of focusing on these contaminants was due to their carcinogenic nature and high concentration in produced water, therefore categorizing them as priority pollutants. The emulsion was constantly agitated and applied vertically to the membrane surface; and permeate passed through the membrane by the aid of the applied pressure. Ultimately the water that is introduced in the dead-end cell would pass through the membrane as permeate. For this experiment, each membrane was tested at different pressure readings of 100, 200 and 300 kPa. Rejection ratio was calculated using Equation (6), where *C*_p_ is the concentration of permeate while *C*_f_ is the concentration of the feed.

(5) $$J_{w}\;=\;\frac{V}{A△t}$$

(6) $$R\;(\%)\;=\;(1-\frac{C_{p}}{C_{f}}\;)\;\times \;100\%$$

## 3. Results and Discussion

### 3.1. Effect of Incorporation of CNT on Membrane EWC and Porosity

Table 1 shows the EWC and porosity results. From the results, it was observed that after adding 0.5% pCNTs, both the EWC and porosity of the fabricated membranes decreased as compared to the membrane with 0% CNTs. However, the EWC and the porosity started increasing considerably with the increase in CNT loading and furthermore after adding fCNTs.

### 3.2. Effect of CNTs on Thermal Stability

**Figure 1:**
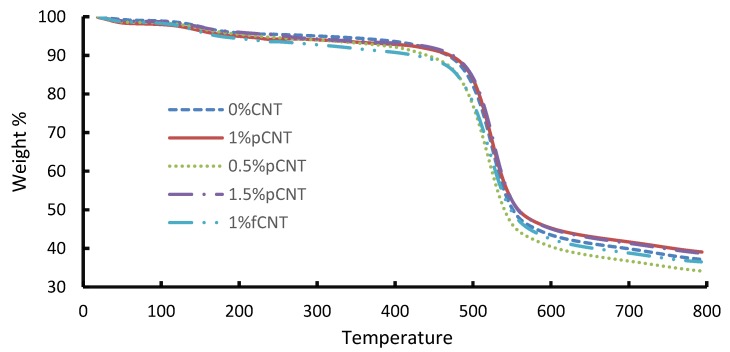
TGA curves of blended polysulfone (PSF)/polyethersulfone (PES) membranes with different CNT concentrations.

**Figure 2:**
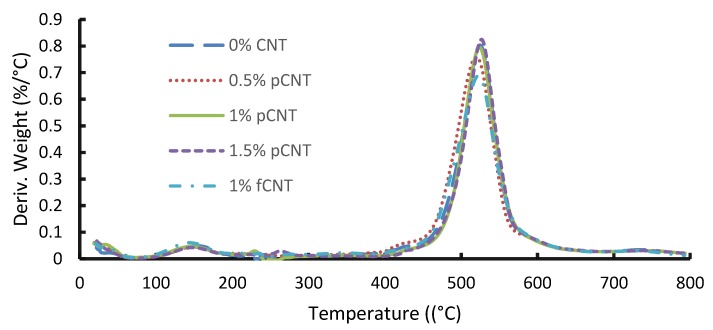
Thermographs of blended PSF/PES membranes with different CNT concentrations.

TGA was used to determine the thermal stability of the nanocomposite membranes. CNT loading had a substantial effect on the thermal stability of polymeric membranes. Due to their unique structural features, CNTs possess excellent thermal properties. The thermal stability of CNTs in general ranges from 720 to 2000 °C depending on their structure [9]. Figure 1 presents the results of the TGA. The slight weight loss observed between 25 and 200 °C is due to the loss of moisture and solvent used during the fabrication of the membrane. Considering Figure 1, the 0 wt. % membrane started degrading at 480 °C. Upon adding pCNTs, with the addition of 0.5 wt. %, the thermal stability decreased, with degradation occurring between 400 °C and 420 °C. This can be attributed to certain defects in the polymer matrix. However, the thermal stability improved started increasing after the addition of 1 wt. % composition, attributable to the high level of compatibility between the CNTs and the polymer matrix. Comparable behavior in terms of thermal stability of membranes containing CNTs has been reported in the literature [10,11]. Upon the incorporation of fCNTs, the thermal stability declined. It has been reported that the functionalization of CNTs tends to reduce the thermal stability of nanocomposite, which is due to the present carboxylic groups on the CNTs that are easily broken at lower temperatures [9]. This could explain the observation when 1 wt. % of fCNTs was added. Figure 2 shows a single high peak, which is an indication of a single-phase separation taking place, thus confirming good compatibility between the blended polymers and CNTs. The shallow peak between 100 and 200 °C represents the loss of solvent (NMP) contained in the membrane, which has a boiling point of 202 °C.

### 3.3. Effect of CNTs on Membrane Tensile Strength

**Figure 3:**
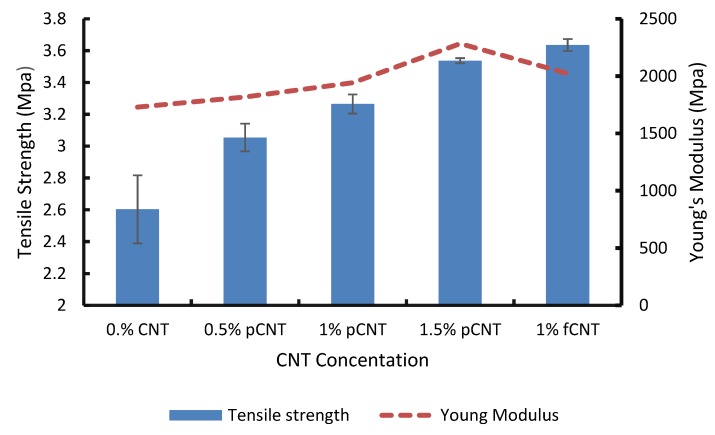
Young’s modulus for CNT/PSF/PES blended membranes.

Incorporation of CNTs has been reported to increase the life span of membranes by enhancing their robustness, making them less susceptible to break during operation [12]. This is due to the strong chemical bonds between carbon atoms found in a single graphene of CNTs. Ideally, pressing on the tip of a nanotube causes it to bend rather than breaking, and usually goes back to its original shape when the pressing force is released [13]. This improves the membrane tensile strength when incorporated in the polymer matrix. Figure 3 summarizes the results obtained from the nano-tensile tests. It is observed that the tensile strength of the membranes increased with an increase in pCNT concentration. The 1.5 wt. % pCNT membrane is the most stable in terms of mechanical strength. The tensile strength of this membrane increased with over 35% as compared to the pristine membrane, thus making it the best membrane with excellent tensile properties in this study. Using the functionalized CNTs in the PSF/PES further maximized the tensile strength of the membrane as expected. From these results, it is seen that embedding CNTs did improve the mechanical strength of the membrane in agreement with the literature [14,15,16]. The improvement in the tensile strength of the membrane could be attributed to the enhanced interaction between the polymer matrix and CNTs [17]. 

**Figure 4:**
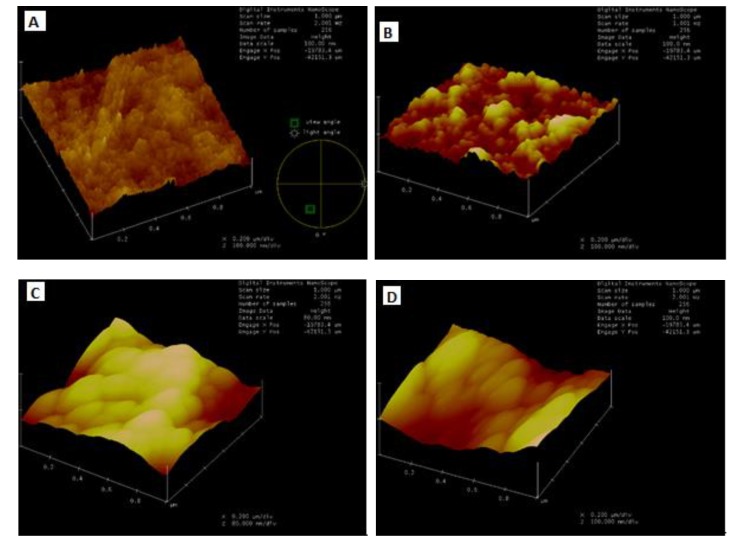

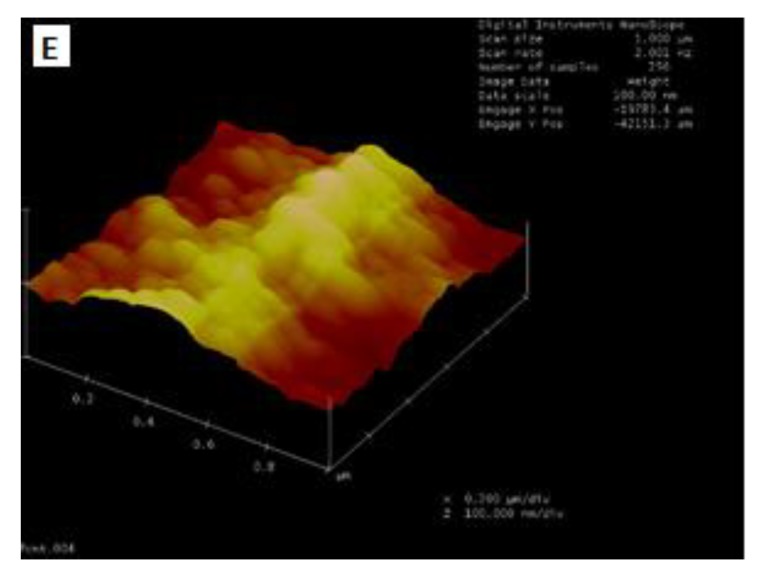
Atomic force microscopy (AFM) 3D surface morphology images of (**A**) 0% CNT, (**B**) 0.5% p-CNT, (**C**) 1% p-CNT, (**D**) 1.5% p-CNT and (**E**) 1% f-CNT.

The same phenomenon was observed for Young’s modulus. The ability of membranes to be stretched under constant pressure influences their industrial applications. As shown in Figure 4, the elasticity and strain of the membrane are dependent on the concentration of the CNTs; it increased with an increase in CNT concentration. From these results, it can be deduced that embedding CNTs has a positive impact on the mechanical properties of the membranes, confirming good interfacial interaction between the CNTs and the blended polymers.

### 3.4. Effect of CNTs on Surface Nature of the Membrane

Generally, PSF and PES are known as hydrophobic polymers that foul easily due to hydrophobic foulant molecules that are driven toward the surface [18]. AFM has become vital equipment for the membrane community when optimizing the fouling properties of membranes used for separation processes [19]. The rougher the surface of the membrane, the more susceptible it is to foul over time. This is because the rough surfaces serve as microhabitats for foulants to flourish and form a cake layer, thus reducing the integrity of the membrane [20]. Therefore, a decrease in surface roughness reduces the ability of contaminants to accumulate on the membrane. For this study, the AFM was used to analyze the effect of CNT loading in the PSF/PES membrane by looking at the peak-to-valley and surface roughness measurements of the prepared membranes. In general, imbedding CNTs alters the properties of the membrane surface, thus generating electrostatic forces between polymer chains [17]. As observed in Table 2, it is shown that the roughness of the membranes decreases with an increase in CNT loading, making the membranes smoother compared to blended polymers. This is due to the hydrophilic nature of CNTs as comparable with the study conducted by Choi [16]. The membrane with fCNT further reduced the roughness as compared to that of pCNTs; this is due to the added carboxylic acid groups on the CNTs surface that reduce the adhesion of fouling agents. Previous literature has reported that during phase inversion, fCNTs tend to travel towards the surface of the membrane, thus inducing the hydrophilic nature of the surface as expected [17]. Additionally, an increase in the hydrophilicity of a membrane provides more opportunity for water to chemically associate with the membrane surface rather than foulants [18]. These results indicate that the composite membranes embedded with CNTs possess smoother surfaces, further improving their anti-biofouling property.

Membranes need to demonstrate non-adhesive properties in order to be considered as robust hydrophilic membranes and be applied in wastewater treatment processes. Increased hydrophilicity of the CNT membranes changes the surface absorption properties, thereby improving the antifouling properties of the membrane [18]. Generally, fouling agents are negatively charged, so adding negative functional groups to the CNTs increases the negative charge density of the membrane, therefore repelling the fouling agents introduced to the membrane during production [21]. The first sign of membrane fouling is decreased flux overtime.

It is evident that the addition of CNT did have an effect on the surface nature of the membrane by altering the charge, hydrophilicity as well as roughness [18]. The same membrane behavior was observed by Zhang et al. [22]. These results confirm the potential of incorporating CNTs to improve the overall performance of commercial membranes by preventing fouling, thereby reducing operational costs and increasing membrane lifespan.

### 3.5. Effect of CNT Loading on Morphology

**Figure 5:**
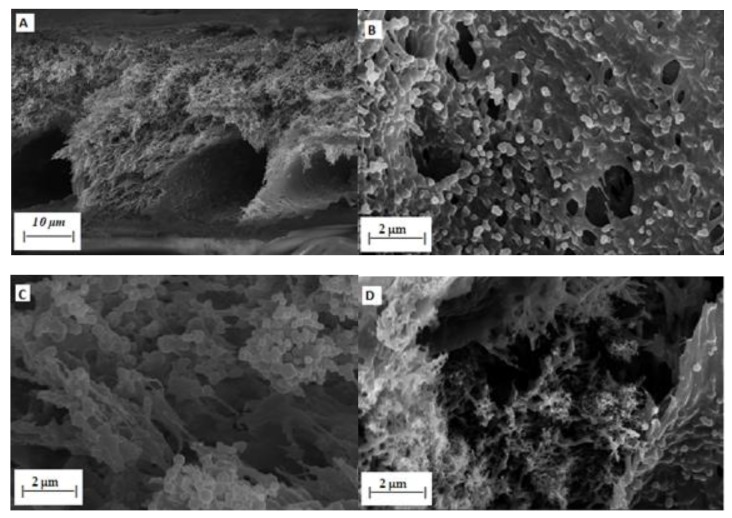

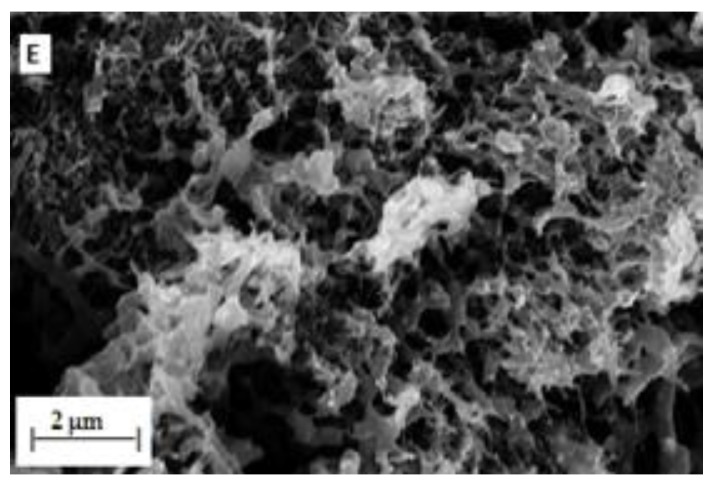
Cross-section SEM images of PSF/PES/CNT membrane (**A**) 25% PSF:75% PES, (**B**) 0.5% pCNT, (**C**) 1% pCNT, (**D**) 1.5% pCNT and (**E**) 1% fCNT.

**Figure 6:**
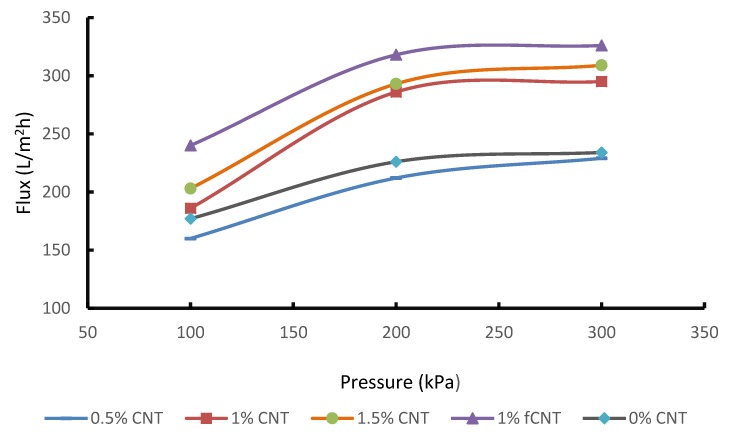
Effect of pressure and CNT concentration on membrane flux.

In order to evaluate the effect of CNT addition on the blended membrane, morphological analysis was conducted using SEM. Images of a cross-sectional view of the membrane were taken. The images were taken at a higher magnification of ×5000. Figure 5 represents a high magnification cross-sectional image of PSF/PES/CNTs membranes. These images show an asymmetric porous structure with CNTs tangled with the polymer. It is clearly seen that the CNTs are arranged in a disorderly manner forming tangled threads. As seen from Figure 5A, the pores are very small and are not visible from the magnification used. However, after the addition of 0.5% pCNTs in Figure 5B, a few CNT strands are visible between the macro-voids. Macro-voids were also formed and increased significantly as the CNT concentration increased. Interfacial defects between the untreated nanoparticles and the polymer solution were expected. It is observed that by increasing the CNT concentration, the agglomeration effect is increased and thus pore formation frequency is increased. These observations are in full agreement with the flux results presented in Figure 6. The membranes with pure CNTs showed multiple localized aggregation which usually happens when the CNTs are not uniformly dispersed. This happens as untreated CNTs tend to agglomerate due to van der Waals forces. Feng et al. [23] reported that incorporating inorganic nanoparticles increases pore sizes when compared to pristine membranes.

### 3.6. Effect of CNT on Pure Water Flux (PWF) and Removal of Phenol and Benzene from Wastewater

The flux of the membrane depends on its pore size distribution and physicochemical properties, especially the hydrophilicity of the surface. The effect of CNT loading on CNT/PSF/PES membranes was investigated through a pure water permeation test. The water flux through the membranes was recorded and compared. As depicted in Figure 6, the membranes flux increased with an increase in pressure because of the increased driving force, which raises the capillary pressure of the membrane hence the pores [24]. CNTs are reported to improve flux due to their hydrophilic walls that are able to interact with water molecules creating a frictionless passage. It is observed that adding 0.5 wt. % pure CNTs to the blended PSF/PES showed a decline in flux as compared to the pristine membrane. The reason for this could be due to the reduced frictional free volume of the polymer matrix. It was only after adding 1 wt. % and 1.5 wt. % pure CNT that the flux was enhanced and increased dramatically as the pure CNT concentration increased reaching a maximum flux of 309 Lm-2h-1 at a pressure of 300 kPa. One membrane was prepared with functionalized CNTs to compare its performance with the pure CNT membrane. Functionalization of CNTs is generally known to influence both the chemical and physical properties of membranes, where the addition of these modified nanoparticles tends to further increase the surface pore sizes or the number of pores [25]. Mehrabadi et al. [17] reported that by functionalizing CNTs, some walls of the pore channels tend to break down, thus forming larger pores due to the accelerated phase separation during the phase inversion process. Nguyen et al. [26] argued that the increase in flux after functionalization is due to hydrogen bonding between water molecules influenced by oxygen elements from the added functional groups, thus forming a thin hydration layer on the membrane surface. This confirms why the flux of the fCNT was higher than that of all pure CNTs with a maximum flux of 326 L/m^2^h at a feed pressure of 300 kPa.

**Figure 7:**
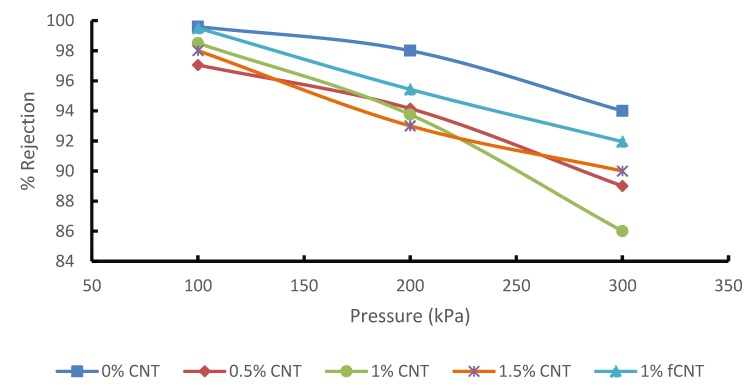
Effect of pressure and CNT concentration on benzene rejection.

**Figure 8:**
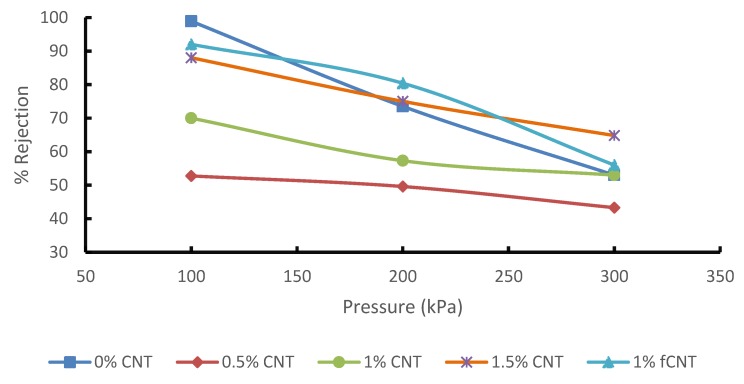
Effect of pressure and CNT concentration on phenol rejection.

The separation performance test was conducted to evaluate the effect of CNT concentration and CNT functionalization on the separation performance of the blended membrane at different pressure readings. However, the performance of these membranes is usually affected by fouling and concentration polarization. For this reason, there have been several studies focused on membrane modification to improve separation performance. These modifications are to enhance and open new avenues for the commercial application of membranes. The addition of CNTs not only influences the membrane flux, but it also has an effect on its selectivity. The effect of CNTs loading on phenol and benzene rejection was investigated during the treatment of synthetic wastewater containing 20 mg/L benzene and 20 mg/L phenol. Figure 7 show the PSF/PES membrane has a higher benzene rejection for both phenol and benzene at than the PSF/PES/CNTs membranes. Figure 8 shows % rejection of phenol, the PSF/PES membrane showed a higher phenol rejection at a lower pressure of 100 kPa, however, the % rejection decreased as the pressure increased. The reason for the low % jerection in membranes with pCNTs could be attributed to poor CNT dispersion and poor compatibility between polymer matrix and CNTs, which results in increased macro voids, creating pores larger than phenol and benzene molecules. The membrane with fCNT showed enhanced % rejection as compared to that of pCNTs for both phenol and benzene, confirming enhanced CNT dispersion as expected after functionalization. These results agree with the analysis obtained from the SEM as illustrated in Figure 5. Even at reduced feed pressure, % rejection of membranes with CNTs tends to decrease with an increase in pCNT loading, which is a result of an increasing number of pores as CNT concentration increases. This also explains the enhanced permeation flux of these membranes as illustrated in Figure 6. These results are comparable to what Phasha reported [14]. A decrease in the selectivity of phenol and benzene was observed as the feed pressure increased; this can be attributed to the increased driving force that pushes phenol and benzene molecules through the membrane pores.

## 4. Conclusions

PSF/PES membrane embedded with pCNTs and fCNTs were prepared successfully via the phase inversion method. These membranes were tested during the treatment of synthetic wastewater containing phenol and benzene. The addition of CNTs enhanced both the porosity and the membranes’ ability to absorb water from 1 wt. % CNT onwards. The flux of the membrane was 309 L/m^2^h for 1.5% pCNT membrane and 326 L/m^2^h for fCNTs membrane. However, as flux increased, % rejection decreased as compared to the pure membrane with no CNTs. This is due to the opening of pores and macro voids observed from the morphological analysis of these membranes. Though the % rejection decreased, all membranes showed acceptable benzene rejection while only two membranes (1.5% pCNT and 1% fCNT) produced the desired results for phenol rejection. The effect of feed pressure on the performance of the membrane was also evaluated. Increase in feed pressure enhanced membrane flux. However, % rejection was inversely proportional to the increasing pressure. The morphological analysis of the membranes obtained from the SEM confirmed the presence of CNTs, with a noticeable agglomeration of CNTs within the membranes. However, the functionalization of the CNTs improved the dispersion of CNTs, attributable to improved interfacial interaction between the polymer and the nanoparticles. The mechanical strength, thermal stability and surface nature of the membrane also improved drastically after adding CNTs, with optimum results obtained from the membrane with fCNTs, which was expected. The roughness of the membrane decreased with an increase in CNT content, thus enhancing the antifouling properties of the membrane. According to the results obtained, reinforcing fCNT in the blended membrane produced a desirable membrane material with good separation performance.

## Figures and Tables

**Table 1:** Effect of carbon nanotube (CNT) loading on ultrafiltration characteristic of membranes.

Membrane	EWC (%)	Porosity (%)
0% CNT	61.58	55.39 ± 5.6
0.5% p-CNT	46.81	33.67 ± 4.8
1% p-CNT	60.17	57.39 ± 2.6
1,5% p-CNT	63.78	58.07 ± 4.2
1% fCNT	68.36	60.89 ± 4.7

**Table 2:** Effect of CNT loading on membrane roughness.

Membrane	Mean Surface Roughness (Ra-nm)	Root Mean Surface Roughness (Rq-nm)
0% CNT	7.160 ± 1.5	9.010 ± 1,6
0.5% p-CNT	3.523 ± 2.1	4.594 ± 2.8
1% p-CNT	3.402 ± 1.9	4.315 ± 2.6
1,5% p-CNT	2.736 ± 2.8	3.572 ± 1.2
1% fCNT	1.865 ± 0.7	2.420 ± 1.7
